# SUTO-Solar Through-Turbulence Open Image Dataset

**DOI:** 10.3390/s22207902

**Published:** 2022-10-17

**Authors:** Adam Popowicz, Valeri Orlov

**Affiliations:** 1 Faculty of Automatic Control, Electronics and Computer Science, Silesian University of Technology, Akademicka 16, 44-100 Gliwice, Poland; 2 Instituto de Astronomia‚ Universidad Nacional Autonoma de Mexico, Av. Universidad 3000, C.U., Coyoacán, Mexico City 04510, Mexico

**Keywords:** turbulence, image datasets, deconvolution, solar images

## Abstract

Imaging through turbulence has been the subject of many research papers in a variety of fields, including defence, astronomy, earth observations, and medicine. The main goal of such research is usually to recover the original, undisturbed image, in which the impact of spatially dependent blurring induced by the phase modulation of the light wavefront is removed. The number of turbulence-disturbed image databases available online is small, and the datasets usually contain repeating types of ground objects (cars, buildings, ships, chessboard patterns). In this article, we present a database of solar images in widely varying turbulence conditions obtained from the SUTO-Solar patrol station recorded over a period of more than a year. The dataset contains image sequences of distinctive yet randomly selected fragments of the solar chromosphere and photosphere. Reference images have been provided with the data using computationally intensive image recovery with the latest multiframe blind deconvolution technique, which is widely accepted in solar imaging. The presented dataset will be extended in the next few years as new image sequences are routinely acquired each sunny day at the SUTO-Solar station.

## 1. Introduction

Imaging through turbulence has been the subject of many research papers in a variety of fields, including defence, astronomy, earth observations, and medicine. The main goal of such research is to recover the original, undisturbed image, in which the impact of spatially dependent blurring induced by the phase modulation of the light wavefront is removed. In many research papers, the authors use a small dataset of image sequences, usually obtained in a limited time frame using their own equipment, in order to prove the superiority of a new approach. Their observations also involve distant objects (e.g., >1 km), while imaging equipment typically includes zoom lenses (e.g., >100 mm focal length) and common CCD cameras. Only a few, and rather limited, datasets are available online.

The work of [1] is a good example of how researchers usually proceed in studies on improving the quality of turbulence-distorted images. For the purpose of verifying the authors’ proposed algorithm, a series of several observations of a ship 2.5 km away was prepared from a 45 m high tower using a 300 mm lens connected to an Allied Vision Mako G-125C 1/3${}^{\prime \prime }$ colour CCD camera. The database did not contain ground-truth images, and the evaluation of the quality of deconvolution results was based solely on a visual assessment (perceptual image quality). This, and similar studies, are usually complemented by simulation data, in which images are degraded according to an assumed model. In this case, the method presented in [2] was adopted.

An example set of images degraded by turbulence was captured during the NATO-RTH40 experiments carried out at White Sands Missile Range [3,4]. The main objective of this research was to test methods for modelling the effects of turbulence on image quality. Conditions for recording a chessboard-like panel from a distance of 1 km were monitored by numerous additional devices (mainly scintillometers) placed on the path between the camera and the target. A fast-motion TV camera was used to obtain data in the visible spectrum of light, and a total of 135 sequences were recorded under different turbulence conditions. Images were taken over several days, during which turbulence conditions varied according to the time of the day. The edges of the images were then analysed, with the set of observed targets being limited to just simple patterns. Another NATO set was captured during the NATO RTO SET 165 trial [5]. In addition to the characterisation experiments refracting a laser beam using a turbulent atmosphere, the researchers collected a series of 200 images of a scene containing a building, a person, and a chessboard pattern, all 7 km away from the observer. This particular dataset was severely degraded by turbulence.

A number of different types of objects were used to create a database of turbulence-degraded images in [6]. Some of the data were simulated images, for which six gas hobs placed between the object and the camera were used. The remaining data were obtained by imaging from distances between 200 m to 1.5 km. The resolution of the images was between 100 × 100 and 1000 × 1000, with 50 to 200 frames per sequence recorded. Detailed information of the observed objects was presented in Table II of the original paper. The main objective of the authors was not to acquire a wide database of various targets under different turbulent conditions, but to acquire only short sequences of a few sample target types in order to verify the improvement in image quality with the proposed algorithm.

Another rather limited dataset was taken during the experiments presented in the work of [7]. The data included three objects: a building, a chimney, and a book. A 200 mm lens was used for imaging, which had, as in the previous work, a resolution between 100 × 100 and 200 × 200 pixels, with 100 frames per sequence recorded. An additional series of 200 frames (227 × 227 pixels) of the Copernicus Crater on the Moon was recorded using a Celestron C14 astronomical telescope and a DMK 31 AF033 CCD camera. The remaining collections of images were used by the authors, included only artificially blurred images.

An Open Turbulent Image Set (OTIS)—a specially constructed set of images affected by turbulence–was presented in [8] and can be found at Zenodo (https://zenodo.org/communities/otis/, accessed on 16 October 2022). This dataset was divided into static and dynamic sequences, while turbulence conditions were divided into weak, moderate, and strong. The observed objects contained mainly fixed image patterns, while the moving object was a remote-controlled model car. In addition to the patterns, a distant building door was also observed. Objects were observed using a GoPro Hero 4 Black camera and a 25 mm f/2.0 lens on a synthetic practice field at the San Diego State University, which ensured that heat from the sun was well reflected, and that air turbulence conditions were created in the path between the camera and the targets. Fixed patterns were accompanied by their images undisturbed by turbulence, while ground-truth binary masks were provided for the moving object sequences.

The main issues with current turbulence-distorted image databases are their scarcity and limited content. They have a small number of images acquired in a limited time frame (only up to a few days) and concern only a small group of objects, frequently reproduced across databases (such as cars, ships, buildings, or chessboard-type patterns). A particular problem is also the lack of ground-truth images provided, which would allow the use of objective image quality measurements (instead, so-called “no-reference” measures have to be adopted). Obviously, obtaining a reference image in distant imaging experiments is often impossible; therefore, there might rather be some suggestions of ground-truth images obtained from state-of-the-art verified restoration techniques. Unfortunately, no such auxiliary data are provided.

In this article, we present a database of solar images obtained from the Silesian University of Technology Observatories (SUTO) Solar patrol station (www.suto.aei.polsl.pl, accessed on 16 October 2022). Recorded over a period of more than a year, the short series of interesting fragments of the solar chromosphere and photosphere represent a new and unprecedented type of data usable by current and future algorithmic solutions. The images contain structures very different from the common objects included in existing databases. Our data were recorded for a wide range of atmospheric and observational conditions (e.g., height of the sun above the horizon). In addition to the turbulence perturbed data, we include the most advanced, validated proposed ground-truth images obtained for solar imaging, as well as computationally intensive, multiframe blind deconvolution (MFBD [9,10,11,12]) solutions. The proposed dataset is available online and will be augmented in the future, since data are routinely registered and downloaded from the SUTO-Solar patrol station.

The main contributions of the research are:

1. A unique set of turbulence-degraded images with accompanying MFBD-based, recovered, high-resolution images;
2. Long-lasting (more than 1 year) observations in varying conditions: temperature, humidity, pressure, elevation of the target;
3. Environmental conditions registered on auxiliary sensors;
4. Small-aperture telescopes together with an off-the-shelf CMOS camera—a relatively low-cost setup suitable for wide utilisation in similar through-turbulence imaging experiments;
5. A range of run-time and postprocessing algorithms specially developed for the experiment: from the selection of image patches containing varying or unusual structures to data cleaning procedures (i.e., series selection)

## 2. Imaging through the Atmosphere

**Figure 1:**
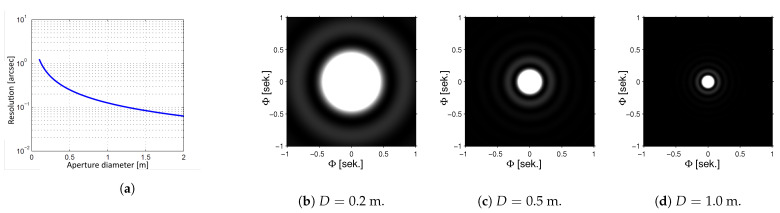
Resolving power of a telescope: (**a**) relationship between resolving power (C) and mirror size (*D*); (**b**), (**c**) and (**d**): sample Airy discs for telescopes with mirror sizes of 0.2 m, 0.5 m and 1.0 m, respectively.

The theoretical resolving power of a telescope is defined through the size of the Airy disc (the image of an ideal distant point source, obtained on a circular aperture) and takes the following form:

(1) C=1.22λ/D,

where C is the resolving power given in radians, λ is the wavelength of the observed light, and *D* is the diameter of the telescope mirror. Figure 1 presents the relationship between resolving power and size of the mirror, and Figure 1b–d present sample Airy discs for three aperture sizes.

Unfortunately, the resolution of the obtained images (especially for long exposure times that are required to registered dim objects) is usually far lower than the theoretical capability of a telescope. This is because turbulence in the Earth’s atmosphere blur the details of the observed objects. The resolution of images registered using even the largest telescopes has been found to be similar to that registered using a telescope with a diameter of only 0.2 m.

**Figure 2:**
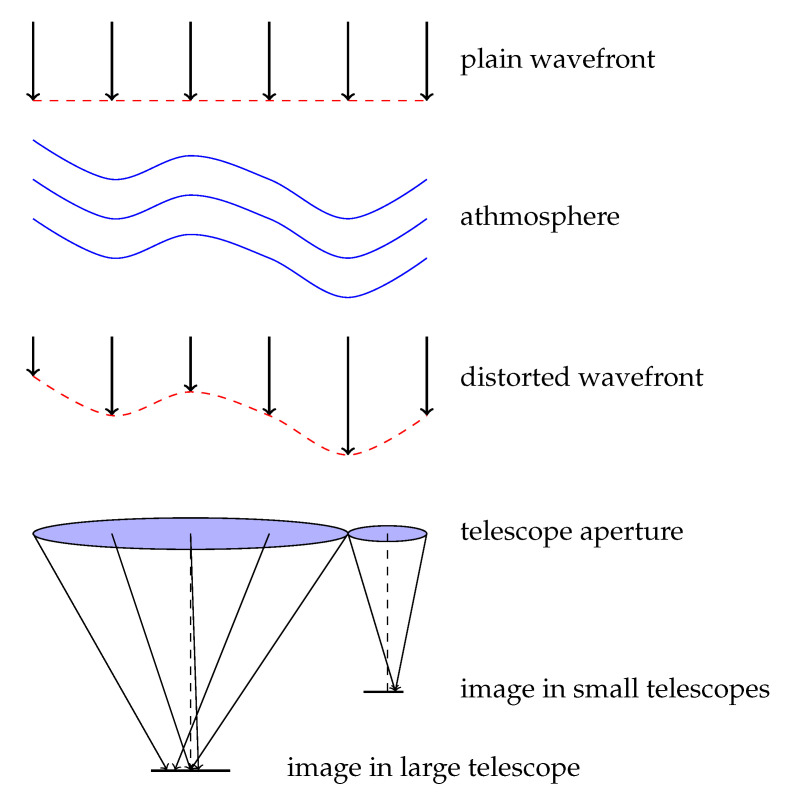
Resolution drop in an image of a point source due to atmospheric turbulence.

**Figure 3:**
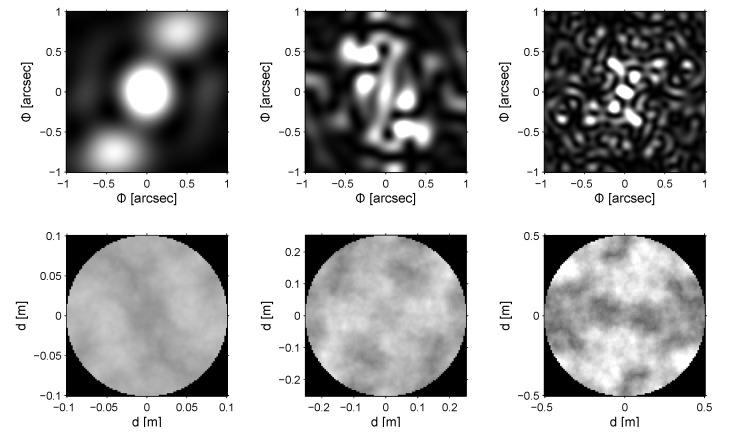
Simulated images (**top**) and the corresponding wavefront distortions (**bottom**) in observations of point sources using telescopes with diameters of 0.2 m, 0.5 m and 1.0 m (Φ—the observed angular area). The images correspond to very short exposure times (i.e., below the time constant of atmospheric coherence). The wavefront images use a greyscale in which the colours white and black correspond to phase shifts of 0 and 2π, respectively.

Figure 2 outlines the issue of wavefront distortion and the drop in resolution that result from atmospheric turbulence. Light coming from a distant object is diffracted when passing through the atmosphere due to a varying refractive index of moving blocks of air. An initially coherent wavefront is distorted, and the obtained image is far from the benchmark Airy disc. Telescopes with a small mirror diameter primarily suffer from the tip–tilt effect, whereas large apertures primarily suffer from speckles. Figure 3 presents sample images from a point source obtained for different apertures under the same atmospheric conditions. The simulation was based on equations derived in [13].

The presented images of distorted point sources can be understood as blurring kernels (or point spread functions, PSFs) that degrade the complex images. Therefore, the image creation process can be expressed as:

(2) $$I_{out}\left(x,y\right)=PSF_{x,y}\left(u,v\right)*I_{in}\left(x,y\right),$$

where $I_{in}\left(x,y\right)$ and $I_{out}\left(x,y\right)$ are images before and after degradation by turbulent medium, $PSF_{x,y}\left(u,v\right)$ is the blurring kernel for the (x,y) position in the image plane (u,v are coordinates in the kernel’s plane), and * denotes the convolution operation. The PSF not only evolves with time but also varies across image plane, which makes the process of image restoration very difficult.

Kolmogorov [14] proposed a model of atmospheric turbulence in 1941 which was later expanded by Fried [15]. The most important conclusions from Kolmogorov and Fried’s research concern three parameters that describe the stability of a turbulent medium:

1. The Fried parameter, $r_{0}$, which denotes the distance in the pupil plane at which the phase shifts in the wavefront amount to an average of 1 radian (under daytime conditions $r_{0}$ = 1–5 cm, whereas the best-located observatories in the world achieve a night-time atmospheric stability of $r_{0}$ = 20 cm);
2. The coherence time, T, or the time constant of the relative stability of a wavefront, which denotes the time for which the wavefront remains unaltered (for visible light, T is up to a few milliseconds, but it can drop well below a millisecond during daytime observations in the visible region);
3. The isoplanatic angle, $\theta_{i}$, which denotes the angular distance of two objects that can be assumed to have a similar distortion (from one to several arc seconds for visible light and near-infrared, respectively). In other words, the PSF variations within the isoplanatic angle are not significant.

All three parameters depend strongly on wavelength. The examples above provide the values for visible light (λ=500 nm), whereas an improvement is obtained by moving towards IR (i.e., all three parameters increase), which is why most high-resolution observations are conducted not in the visible spectrum, but in the near-infrared region. Popular high-resolution imaging bands include the J and K ranges, which correspond to wavelengths of 1220 nm and 2190 nm, respectively.

For daytime telescopic observations of solar activity, the atmospheric conditions are much worse than at night. This results in a lower coherence time (by several factors) and a smaller Fried parameter $r_{0}$. Fortunately, the solar observations with small telescopes are affected by the atmosphere similarly to large telescopes working in night-time conditions. This is because a large part of the image degradation for small solar telescopes observing wide fields of view results from the so-called ground layer, i.e., the layer of atmosphere close to the ground (see, e.g., [16,17,18]). The isoplanatic angle for the contribution of the turbulent ground layer is many times larger than for high-altitude layers because of the close proximity to the telescope entrance pupil. The coherence time is also much larger, reaching tens of milliseconds, because of the same origin. The phenomenon of ground-layer turbulence is actually the main contributor to the degradation of image data presented in this study.

## 3. Experiment

### 3.1. SUTO-Solar Patrol Station

SUTO-Solar is the only station operating on any clear day to register the solar disk in Poland. The station was installed by the scientific group at SUTO (Silesian University of Technology Observatories) in June 2021. Its main task is to provide images of the full solar disk in the H-alpha band (656.28 nm, band FWHM 0.05 nm) and in the so-called broadband solar continuum around 540 nm (band FWHM 10 nm). Current and archived data, both in the form of (1) appropriately formatted raw FITS files, (2) compressed JPEGs, and (3) video animations are available on the SUTO group website under the “Solar Observations” tab.

The solar station uses two telescopes; the first is a specialised Lunt 60 solar telescope for imaging the solar chromosphere in the hydrogen line 656.3 nm and is additionally equipped with a filter that narrows the light transmission to a width of 0.05 nm (so-called double-stack module). The observations of the photosphere are made with a Sky-Watcher ED80 refractor and Baader Solar Continuum filter at 540 nm (10 nm bandwidth). The technical details of the optical equipment used are given in Table 1.

The imaging devices on both telescopes are the same monochrome CMOS cameras: the ASI ZWO 178 MM. They have the advantage of a relatively low price yet a high sensitivity and small pixel size that allows them to be combined with telescopes with moderate focal lengths. This combination of SUTO-Solar telescopes results in a resolution of about one angular second per pixel. This is more than twice as low as the theoretical diffraction resolution of the instruments, but it means the potential of the optics is fully exploited. More details on the characteristics of the utilised sensors are presented in Table 2.

Additional sensors were connected to the telescopes to monitor the current temperature, humidity around the optics, and atmospheric pressure. The popular DHT11, BMP280, and Si70 sensors were used for this purpose. The first of them was attached directly on the telescopes’ housing, while the other two were located on the mount. The sensors were added to enrich the collected images with additional environmental data, which could be utilised in further experiments. The details of the used sensors are given in Table 3.

### 3.2. High-Resolution Experiment

In addition to typical observations of the full solar disk at a refresh rate of every 10 s or so, the SUTO-Solar telescopes take images of selected sections of the solar region for high-resolution experiments every 5 min. For this purpose, the thresholded (arbitrary value of 5000 ADU (ADU—analogue-to-digital units; 1 ADU = 1e ${}^{-}$ in utilized ZWO183MM cameras) sun disk area was divided into a grid of 100 × 100-pixel patches with a random offset of the grid in both axes (from 0 to 100 pixels). Only patches included entirely in the thresholded solar disk were considered in next steps. Starting from 16 July 2022, we changed the size of patches to 200 × 200 pixels and added a patch registration from the photospheric telescope, due to the establishment of a better internet connection and increased capabilities of data transfer

**Figure 4:**
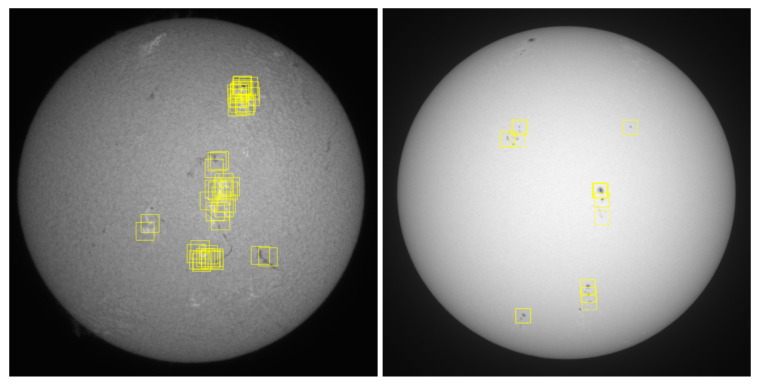
Sample solar disk in H-alpha (**left**) and in solar continuum (**right**) with marked selected regions in 50 consecutive runs of our pseudorandom patch selection procedure.

**Figure 5:**
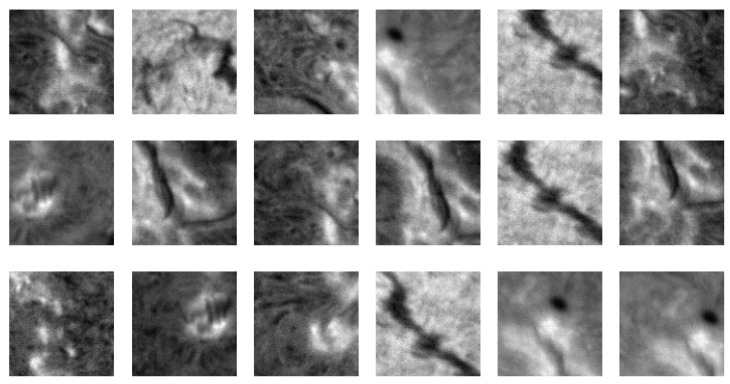
Patches selected in the first 15 runs of the adopted procedure performed on a chromospheric image presented on the left side of Figure 4.

In the image divided in this way, the variance of each patch was determined before the area with the highest variance was selected. Due to the random shift of the grid, the content of the chosen area changed randomly with successive imaging over a 5-minute cycle. Moreover, thanks to the adopted pseudorandom procedure, the areas of solar disk from which the patches were picked could be far apart. Examples of the outcomes of 50 consecutive runs of this procedure on a sample single chromospheric image registered on 20 July 2022 are presented on the left side of Figure 4. The first 15 selected samples are exposed in Figure 5.

The location of the patches in the broadband solar continuum at 540 nm with ED80 refractor required a different approach to patch selection due to the significantly lower number of interesting regions. Most of the photosphere is only covered with granulation, without any interesting features. Only the so-called photospheric spots exhibit a larger intensity variance. The number of spots is usually small, and only a few of them appear. Since the spots are also relatively small, the procedure adopted for chromospheric imaging would result in the selection of the same spot each time (only shifted randomly in both axis). Therefore, we had to slightly modify the algorithm so that random spots were selected each time.

**Figure 6:**
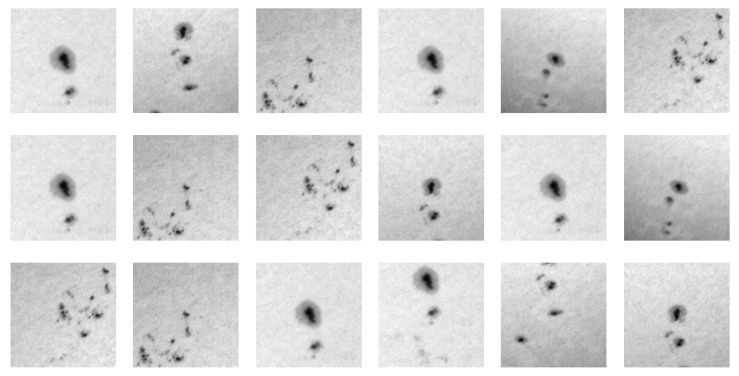
Patches selected in the first 15 runs of the adopted procedure performed on the continuum image presented on the right side of Figure 4.

After thresholding the solar disk (arbitrary value of 10,000 ADU), applying the randomly shifted 200 × 200-pixel grid, and measuring the intensity variance in each box in the grid, we calculated the standard deviation ($\sigma_{v}$) of the obtained variances. Next, we detected possible outlying patches for which the variance was larger than $3\sigma_{v}$, and assumed them as patches that included the sunspots. We randomly picked one of them and additionally applied centring using the centre of gravity. This way, the patch was centred around the sunspot area (however, for very small spots and large fluctuations of granulation in the background, the objects might still be offset). If no patch exceeded $3\sigma_{v}$, there was probably no spot in the photosphere, and the patch with the largest variance was taken since it was suspected to include some larger fluctuations of granulation. Examples of consecutive selections of patches in the continuum are presented in Figure 6.

As the patch location was selected in both telescopes independently, the cameras acquired 100 registrations of images sequentially (starting from 16 July 2022, we increased the sequence size to 200 frames). The selected frame rate was approximately 10 frames per second, and the exposure time was 30 ms. The utilised frame rate guaranteed that each consecutive image was significantly different from the previous one (the time difference between two images, 50 ms, was larger than the expected coherence time of daytime observations, even for the ground layer, so every image was blurred differently). On the other hand, the 30 ms exposure time was adopted as a minimum for obtaining visually satisfactory light signal in observations in a very narrow H-alpha bandwidth of 0.05 nm. The ED80 refractor used for continuum observations required a solar attenuation filter/foil that reduced the heat in the telescope. Eventually, the amount of light falling on the sensor was approximately similar in both telescopes, and thus the exposure times were also the same.

## 4. Data Processing and Dissemination

### 4.1. Series Selection

The data obtained by SUTO-Solar had to be preprocessed for further use since two problems arose. The first was the presence of passing clouds during a series registration. Unfortunately, it was impossible to determine whether the frame would be obscured by clouds during the registration of an image series or not. The SUTO-Solar station has some mechanisms to detect clouds in patrol, full-disk images so that the telescope is prevented from acquiring observations during the next 30 s. However, it is frequent for conditions to be, first, favourable when assessing cloud cover and, second, to change significantly during a series registration.

The second problem identified was the need to remove a series of images of only granulation in the solar photosphere. Such recordings take place when there are no spots on the solar disk. Images of this type, due to their repeatability and near-identity, were removed from the dataset. It is worth mentioning that photospheric granulation is present in all photospheric images, since it is the background of sunspot structures.

**Figure 7:**
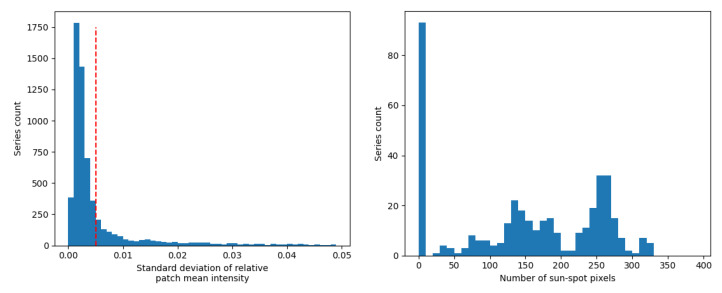
Distribution of relative standard deviations of mean intensities (**left**) and the detected number of sun-spot pixels (**right**) for the series acquired by SUTO-Solar. Red dashed line in the left plot indicates the threshold for series affected by cloud cover.

To detect series disturbed by passing clouds, we analysed the standard deviations of the mean intensities of images in a series. The mean intensity was calculated for each image in a series, and then from such a vector of means, a standard deviation was calculated. This procedure was repeated for all series, for both photospheric and chromospheric datasets. A histogram showing the calculated relative standard deviations is presented on the left side of Figure 7. We arbitrarily selected a value of 0.05 as a threshold. Series showing higher values were removed due to their high distortion by the cloud cover.

To detect the series with only granulation (no sunspots), we adopted a procedure to find pixels possibly lying within sun spot areas. First, we calculated the median intensity ($I_{med}$) within the patch across the whole time series (i.e., the median of all pixels in all images in a series). Then, we calculated the standard deviation ($\sigma_{std}$) the same way (i.e., through all pixels in all images in a series). Next, we found the pixels whose intensity was lower than $I_{med}-5\sigma_{std}$. The number of pixels divided by the number of images in series for the photospheric dataset (cleaned from the series affected by clouds) is presented on the right side of Figure 7. As it can be seen, almost 90 series were obtained possibly without any sunspots—the number of detected pixels was 0, and thus these observations were removed from our dataset.

### 4.2. MFBD Processing

It is not possible to obtain a perfect, undisturbed reference image for each image sequence. Of course, there are images of the solar disc acquired from other, larger telescopes (even orbital), characterised by a higher resolution. Unfortunately, they are obtained by different optical instruments with different characteristics (e.g., the telescopes observing in H-alpha differ in their bandwidth, not to mention the different aberrations distribution, etc.). Such images could not be considered as reference data to our dataset.

In order not to leave the base without any reference data, we decided to prepare high-resolution “pseudo-reference” data using state-of-the-art tools widely accepted in solar astronomy. We used the multiobject multiframe blind deconvolution (MOMFBD) approach prepared by authors of [19], and made available as a REDUX open-source code (https://dubshen.astro.su.se/wiki/index.php/Redux, accessed on 16 October 2022). Although this solution was utilised mostly on large solar telescopes, its universality and rich adjustable parameters allow it to be used on small wide-field solar instruments as well.

Each series of photospheric or chromospheric images was processed by REDUX using the parameters obtained by consulting with its authors. Considering the significant impact of the ground layer, we adopted a large value of isoplanatic patch of approximately 1.5 arcmin. This resulted in an almost 100×100-pixel area which was treated as homogeneously disturbed by blurring kernels. The 100×100 patches (data collected before 16 July 2022) were therefore processed without image splitting in MFBD, while the 200×200 ones (data collected after 16 July 2022) had to be split by REDUX into 2×2 subpatches. The selection of the large isoplanatic patch was also dictated by the low resolution of images, as well as the need for working with subpatches with sides at least several tens of pixels (to minimise artefacts appearing after joining deconvolved subpatches).

The results of REDUX were images smaller than the original versions since REDUX requires some space on the image edges to apply deshifting (compensating for the tip–tilt effect, as well as possible telescope shaking). Eventually, each 100×100-pixel series received a 70×70 MFBD output, while the 200×200-pixel series resulted in a 177×177 outcome. The results were centred relatively to the input series.

We ran MFBD on a 40-core CPU cluster (using all CPUs) with an average processing time for the 100×100-pixel 100 images series at 20 s, and up to a minute for the 200×200-pixel 200 images series. Therefore, the MFBD algorithm required intensive calculations using a multithread approach and was definitely not applicable for real-time image reconstruction even with large computational capabilities.

**Figure 8:**
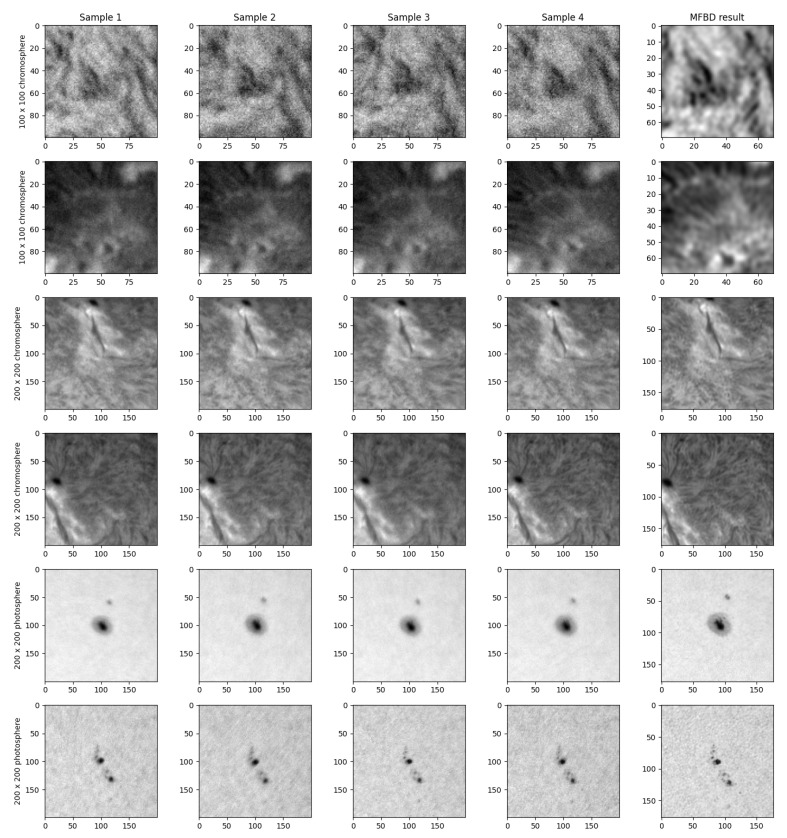
Sample results from MFBD processing together with the first 4 samples of image series. Data from 100×100- and 200×200-pixel formats, for both chromosphere and photosphere.

Example results from the MFBD processing, together with the first four samples from the series, are presented in Figure 8. Rows 1 and 2 show 100×100 chromospheric data, rows 3 and 4 show 200×200 chromospheric data, and rows 5 and 6 show 200×200 photospheric data. Not only is the resolution improved, but also the signal-to-noise ratio is visibly elevated by MFBD processing (especially evident in the first two examples).

We wanted to draw explicit attention to the fact that the data received from MFBD was only an attempt to recover the original image undisturbed by turbulence. These results should be treated with caution, and used mainly as a comparison, and not as target images. Interestingly, some imaging series were taken under very turbulent conditions, and the MFBD outcome was also visibly blurred. Meanwhile, a single raw, unprocessed image of the same content taken under favourable atmospheric conditions (e.g., just after dawn) may have a much better effective resolution.

### 4.3. Dataset Dissemination

The SUTO-Solar through-turbulence open image dataset (SUTO-Solar-TTOD) is publicly available on the Zenodo webpage at https://doi.org/10.5281/zenodo.7064324 (accessed on 16 October 2022), where all the files are archived together. The naming scheme for the FITS files with series of raw images is as follows: *type_pix_number.fits*, where *type* is either *chromosphere* or *continuum*; *pix* is either *100p* or *200p*; and number is a five-digit number padded with leading zeros (e.g., 01023). The MFBD results are provided with the same name and additional *_MFBD* suffix. The FITS raw files have extended headers with all the additional information about the conditions of imaging. The positions in the header with accompanying explanations are provided in Table 4. The total number of files is 3960 series for chromosphere in the 100×100-pixel format (100 images in a single series), 700 series for chromosphere in the 200×200-pixel format (200 images in a single series), and 424 series for continuum in the 200×200-pixel format (200 images in a single series).

## 5. Summary

Removing the impact of turbulence is an isotonic aspect of digital imaging. Although many methods exist, there are no rich datasets available to the public that could become benchmark databases for, firstly, objective comparisons of available algorithms, but secondly for the training of machine-learning-based algorithms. In addition, the available datasets mainly include similar types of terrestrial objects such as distant buildings, ships, towers, chessboard patterns, etc., observed only during a short period.

In this paper, we presented a database of turbulent images built on the basis of more than one year of observations using the solar telescopes of the SUTO-Solar station. The images recorded show fragments of the chromosphere and the solar photosphere, objects hitherto unseen in current available databases. The range of observations covered more than a year of the observatory’s operation, from morning to evening, so that the data set included images in both exceptionally favourable and unfavourable turbulence conditions, additionally modulated by different solar altitudes above the horizon.

As part of future work, we plan to increase the capacity of our database in the next years of SUTO-Solar’s work. There is also a plan to expand the station to include night-time imaging to record objects such as the moon, planets, bright stars, and bright deep-sky objects (e.g., planetary nebulae).

## Figures and Tables

**Table 1:** SUTO-Solar telescopes.

	Lunt 60 mm	Sky Watcher 80 mm
Target	Chromosphere	Photosphere
Aperture	50 mm	80 mm
Focal length	500 mm	600 mm
Resolution	3.27${}^{\prime \prime }$	2.05${}^{\prime \prime }$
(theoretical)		
Wavelength λ	656.3 nm	540 nm
Band FWHM	0.05 nm	10 nm

**Table 2:** SUTO-Solar cameras.

	ASI ZWO183MM	QHY 5L-II
Target	Science images	Guiding
Pixels	5496 × 3672	1280 × 960
Pixel size	2.4 µm	3.4 µm
Bit resolution	12-bit	8-bit
Readout noise	1.5e ${}^{-}$	4e ${}^{-}$
Peak quantum efficiency	84%	74%
Sampling	0.99${}^{\prime \prime }$/0.83${}^{\prime \prime }$	64.5${}^{\prime \prime }$

**Table 3:** SUTO-Solar environmental sensors.

	BMP280	Si7021	DHT11
Target	Pressure	Temp./Hum.	Temp./Hum.
	(outdoor)	(outdoor)	(optics)
Range	300–1100 hPa	−40∼+125 ${}^{\circ }$ C	−20∼+60 ${}^{\circ }$ C
		0∼80% RH	5∼95% RH
Accuracy	1 hPa	0.4 ${}^{\circ }$ C	2 ${}^{\circ }$ C
		3% RH	4% RH

**Table 4:** Description of headers in raw FITS files.

Header Name	Description	Possible Values
SIMPLE	Conforms to FITS standard	T
BITPIX	Data type—number of bits	16
NAXIS	Number of axes of data	3
NAXIS1	Number of pixels in X-direction	“100” or “200”
NAXIS2	Number of pixels in Y-direction	“100” or “200”
NAXIS3	Number of images in series	“100” or “200”
DATE-OBS	Date of observations	e.g., “2021-08-15T09:18:16”
CREATOR	Utilised software	“Python Sequencer (by Adam Popowicz)”
INSTRUME	Telescope and camera	“Lunt60 ASIZWO183MM”
		or “ED80 ASIZWO183MM”
OBSERVER	Observer	“Adam Popowicz”
SITENAME	Name of observing site	“Kotulin”
SITEELEV	Elevations of observing site	“250”
SITELAT	Latitude of observing site	“+50d23m59s”
SITELONG	Longitude of observing site	“+18d37m59s”
EXPOSURE	Exposure time in microseconds	“10,000” or “30,000”
TELESCOP	Telescope mount	“EQMOD HEQ5/6”
RA	Straightness of target (Sun) in degrees	e.g., “144.8291841110412”
DEC	Declination of target (Sun) in degrees	e.g., “14.02033310137856”
OBJCTRA	Straightness of target (Sun) in h-m-s format	e.g., “09h39m19.0042s”
OBJCTDEC	Declination of target (Sun) in d-m-s format	e.g., “+14d01m13.1992s”
AIRMASS	Airmass to target	e.g., “1.359362702015252”
OBJCTALT	Altitude of target (Sun)	e.g., “47.36122476834755”
CENTALT	Same as OBJCTALT	e.g., “47.36122476834755”
ENVDATA	Environmental data from sensors	e.g., “T1 [C] = 32.00; H1 [%] = 37.00; T2 [C] = 34.00;
		H2 [%] = 24.00; T3 [C] = 53.91; P3 [Pa] = 98,673.74”
PIERSIDE	Side of telescope pier	“pierWest” or “pierEast”

## Data Availability

Data reported in this study is publicly availbe at Zenodo page: https://doi.org/10.5281/zenodo.7064324.
